# Device-Associated Nosocomial Infections in Intensive Care Units at Al-Ahsa Hospitals, Saudi Arabia

**DOI:** 10.7759/cureus.50187

**Published:** 2023-12-08

**Authors:** Essa AlSaleh, Balajis Naik, Ali M AlSaleh

**Affiliations:** 1 Infection Control Department, Directorate of Health Affairs, Al-Ahsa, SAU; 2 Infection Control, Al-Ahsa Health Cluster, Ministry of Health Holdings, Al-Ahsa, SAU; 3 Medicine, Arabian Gulf University, Manamah, BHR

**Keywords:** healthcare-associated infection, saudi arabia, icu (intensive care unit), ventilator-associated pneumonia (vap), cauti, clabsi, alahsa, device-associated infection

## Abstract

Introduction

Patients admitted to intensive care units (ICU), especially those with devices used to support their condition, are at a higher risk of getting healthcare-associated infections (HAIs). The aim of the present study was to analyze the surveillance data and assess the device-associated infection (DAI) rates such as central line-associated blood-stream infection (CLABSI), catheter-associated urinary tract infection (CAUTI), ventilator-associated pneumonia (VAP) and ventilator-associated event (VAE) in ICUs of the Ministry of Health (MoH) hospitals in Al-Ahsa region.

Methodology

The study was conducted retrospectively using the surveillance data of governmental hospitals’ intensive care units in the Al-Ahsa region. The surveillance data was collected from 10 ICUs at six MoH hospitals in the Al-Ahsa region during the year 2022. The data from the participating hospitals was entered into the Health Electronic Surveillance Network (HESN) plus program by trained infection prevention control practitioners of the respective hospitals.

Results

An overall CLABSI rate of 4.29 per 1000 central line days was reported during the study period. The CAUTI rate was 0.55 with a range from 0 to 1.29 cases per 1000 urinary catheter days. VAP rate ranged from 0.33 to 2.21 cases per 1000 ventilator days (average of 1.17). The study reported VAE only for the adult medical-surgical ICU (3.36 per 1000 ventilator days).

Conclusion

The present study revealed that the most common DAIs in the Al-Ahsa region are CLABSI and CAUTI. DAI rates generated from this study may be used as benchmarks for regional hospitals. An educational program regarding the prevention and control of DAIs targeting all healthcare workers, especially ICU staff, has to be done in the Al-Ahsa region.

## Introduction

Healthcare-associated infections (HAIs) are nosocomial infections that are not present or incubated at admission and are acquired after admission to the hospital by 48 hours [1]. The safety of patients admitted to healthcare facilities is threatened by HAIs, which can lead to increased costs, morbidity, and mortality [2]. Several factors contribute to HAIs, such as patient-related factors and healthcare systems-related factors, which include surgical procedures, invasive devices, and antibiotics [3]. Moreover, patients admitted to intensive care units (ICUs) are at a higher risk of acquiring HAIs [4,5].

Nearly 5-10% of patients admitted to ICUs reported HAIs in developed countries, while the rate was 50% higher in developing countries [4]. The World Health Organization estimates the worldwide burden of HAIs between 7% and 12% [6]. Many of the HAIs related to devices are caused by multidrug-resistant organisms [7].

Patients admitted to the ICU often undergo invasive procedures, diagnostic and therapeutic procedural management, and life-saving device support such as endotracheal intubation for mechanical ventilation and intravascular and urinary catheters. If recommended care is not followed, device-associated HAIs (DA-HAIs) can occur [6,8,9]. Device-associated infections (DAIs) affect 24.3-27.6% of patients admitted to the ICU [10].

Globally, several studies have described DAIs in ICUs, which include central line-associated bloodstream infection (CLABSI), catheter-associated urinary tract infection (CAUTI), and ventilator-associated pneumonia (VAP) [8,11,12]. The surveillance system is an important tool in determining health problems and prioritizing control measures that lead to patient safety [2,4]. Moreover, continuous HAI surveillance in Western countries has helped to decrease infection rates [13,14]. However, each country should investigate the effectiveness of surveillance on HAIs individually. In Saudi Arabia, the General Directorate of Infection Prevention and Control (GDIPC) at the Ministry of Health (MoH) in cooperation with the Health Electronic Surveillance Network (HESN) established a surveillance program for the identification of HAIs in ICUs with specific inclusion criteria for healthcare facilities to be included in the final surveillance system [15]. Critical care units of MoH hospitals in the Al-Ahsa region are part of the Saudi national HAI surveillance system. HAI surveillance enables healthcare facilities to detect trends and determine the effectiveness of prevention and control programs [16]. Although the surveillance system in the Al-Ahsa region has been operating for a long time, the researchers understand that there has not been research to date that has analyzed the surveillance data. There is a literature gap about DAIs in the Al-Ahsa region of Saudi Arabia. There is one published national HAI surveillance report on DAIs at the national level without regional stratification [15]. Therefore, the present study aimed to analyze the surveillance data, assess the DAI rates (CLABSI, CAUTI, and VAP) in ICUs of MoH hospitals in the Al-Ahsa region with the following specific objectives: to determine the rate of CLABSI in Al-Ahsa hospitals, to find out the rate of CAUTI in Al-Ahsa hospitals, to conclude the rate of VAP/ventilator-associated event (VAE) in Al-Ahsa hospitals for the year 2022.

## Materials and methods

Setting and surveillance

The study was conducted retrospectively using the surveillance data of governmental hospitals’ intensive care units in the Al-Ahsa region. The surveillance data was collected from 10 ICUs at six MoH hospitals in the Al-Ahsa region during the year 2022. Each hospital has an infection control team consisting of an infection prevention and control practitioner (IPC) (surveillance nurse), an infection control physician, and other supportive staff. The IPC nurse is responsible for surveillance in each hospital and has at least two years of experience and training in surveillance. Moreover, each hospital had a clinical microbiologist who tested and identified clinical isolates using standard microbiological methods.

The data from the participating hospitals was entered into the HESN-plus program by trained infection prevention control practitioners of the respective hospitals. The data were collected from four types of ICUs: adult medical-surgical, pediatric medical-surgical, neonatal, and cardiac ICUs. The surveillance system collected device-associated HAIs, which included CLABSI, CAUTI, VAP, and VAE. The national surveillance methods used the CDC's National Healthcare Safety Network (NHSN) device-associated HAI definitions and methods [17]. The rates of device-associated HAIs were compared with regional and international benchmarks, such as the Gulf Cooperation Council (GCC), NHSN, and International Nosocomial Infection Control Consortium (INICC) reports.

Data collection

Data was collected from six government hospitals: four general hospitals and two specialized hospitals (cardiac centers and maternity and children's hospitals). These hospitals were enrolled in the national surveillance system (HESN-plus). The selected institutes were accredited by the Saudi Central Board of Healthcare Institutions. Data was received from the hospitals without a patient identifier. These six hospitals are the only hospitals mandated to report DAIs to MoH under HESN-plus.

Surveillance data analysis

The surveillance system data collected over one year from January to December 2022 was analyzed using SPSS software version 22 (IBM Corp., Armonk, NY, USA). All patients notified with DAI-HAIs were included in the analysis. The DAI rates were calculated by dividing the number of events over the device days by 1000. Device-associated HAI rates are presented as “per 1000 device-days."

Ethical considerations

The de-identified surveillance data was analyzed by an independent researcher who does not work in the surveillance system. The study was approved by the Institutional Review Board (IRB) at King Fahad Hofuf Hospital (H-05-HS-065).

## Results

Facility characteristics

The majority of hospitals (four; 66.66%) were general hospitals. There was one cardiac hospital (16.66%) and one maternal and child hospital (16.66%). Three (50%) hospitals had fewer than 200 beds, while one-third of hospitals (two; 33.33%) had 201-500 beds. The ICU beds were 197 in all hospitals. The adult medical-surgical ICU constituted five (50%) of all ICU beds, followed by neonatal (two; 20%) and pediatric medical-surgical ICU (two; 20%). However, ICU bed capacity was higher in neonatal ICUs (89; 49.7%), followed by adult medical-surgical ICUs (72; 36.5%) (Table 1).

**Table 1:** Characteristics of hospitals included in the surveillance (2022) ICU: intensive care unit

Hospital type	Number of hospitals	% of total
General/central hospitals	4	66.66
Maternal and Children Hospitals	1	16.66
Cardiac centers	1	16.66
Total	6	100
Hospital bed capacity		
	3	50
201–500	2	33.33
501–1000	1	16.66
Total	6	100
ICU type		
Adult Medical Surgical	5	50
Neonatal	2	20
Pediatric medical-surgical	2	20
Medical cardiac	1	10
Total	10	100
ICU bed capacity		
Adult Medical-surgical	72	36.5
Neonatal	89	49.7
Pediatric medical-surgical	26	13.2
Medical cardiac	10	5.1
Total	197	100

The total patients’ days for all patients admitted to ICUs during the study period were 43,641 days, while the total device days were 42,055 device days. These device days resulted in 99 DAIs, with an infection rate of 2.35 DAIs per 1000 patient days. The majority of DAIs (56; 56.5%) occurred in the bloodstream, followed by the lung (26; 26.26%). A total of 42,055 device days were used for calculations, with stratified device days for each of the DAIs (Table 2).

**Table 2:** Events and denominators used in calculating device-associated infection rates (2022) DAI: device-associated infection, CLABSI: central line-associated bloodstream infection, CAUTI: catheter-associated urinary tract infection, VAP: ventilator-associated pneumonia, VAE: ventilator-associated event

Infection	Type of device	Total Device days	Number of events (%)	DAI Rate
CLABSI	Central line	13042	56 (56.5)	4.29
CAUTI	Urinary catheter	14629	8 (8.08)	0.55
VAP	Ventilator	6642	9 (9.09)	1.17
VAE	Ventilator	7742	26 (26.26)	3.15

CLABSI

A total of 13,042 central line days were monitored for the development of CLABSI events in all ICU-admitted patients. An overall of 4.29 CLABSI events per 1000 central line days were reported during the study period. The CLABSI rate ranged from 2.54 to 8.86 per 1000 central line days. The highest CLABSI rate was reported in neonatal ICU and pediatric medical-surgical ICU (8.86 and 7.36 per 1000 central line days, respectively); while the lowest rate was in adult medical-surgical ICU at 2.54 per 1000 central line days (Table 3).

**Table 3:** CLABSI rates by type of ICU (2022) ICU: intensive care unit, CLABSI: central line-associated bloodstream infection

ICU type	CLABSI events	Central line days	CLABSI rate
Adult Medical Surgical	23	9043	2.54
Neonatal	21	2370	8.86
Pediatric Medical Surgical	12	1629	7.36
Total	56	13042	4.29

CAUTI

The CAUTI rates ranged widely from 0 to 1.29 cases per 1000 catheter days (the overall rate was 0.55). The total number of urinary catheter days that were monitored for the development of CAUTIs in all ICUs was 14,629 urinary catheter days. The pediatric medical-surgical ICU showed the highest CAUTI rate (1.29/1000 catheter days), while the neonatal ICU and adult medical-surgical ICU (0 and 0.52) showed the lowest, respectively (Table 4).

**Table 4:** CAUTI rates by type of ICU (2022) ICU: intensive care unit, CAUTI: catheter-associated urinary tract infection

ICU type	CAUTI events	Urinary Catheter days	CAUTI rate
Adult Medical Surgical	7	13417	0.52
Neonatal	0	436	0
Pediatric Medical Surgical	1	776	1.29
Total	8	14629	0.55

VAP

The total ventilated days in all ICUs were 6642 ventilator days. The overall VAP rate ranged from 0.33 to 2.21 cases per 1000 ventilator days (overall rate: 1.17 per 1000 ventilator days). The highest VAP rate was in the pediatric medical-surgical ICU (2.21 per 1000 ventilator days), while the neonatal ICU showed the lowest rate (0.33 per 1000 ventilator days) (Table 5).

**Table 5:** VAP rates by type of ICU (2022) ICU: intensive care unit, VAP: ventilator-associated pneumonia

ICU type	VAP events	Ventilator days	VAP rate
Neonatal	1	3017	0.33
Pediatric Medical Surgical	8	3625	2.21
Total	9	6642	1.17

VAE

The VAE rates during the study period were reported from only the adult medical-surgical ICU and were 3.36 per 1000 ventilator days. The total number of ventilator days monitored for the development of VAE in all ICUs was 7742 ventilator days (Table 6).

**Table 6:** VAE rates by type of ICU (2022) ICU: intensive care unit, VAE: ventilator-associated events

ICU type	VAE events	Ventilator days	VAE rate
Adult Medical Surgical	26	7742	3.36

Table 7 shows the benchmarks of Al-Ahsa hospitals (regional) with the national rates of device-associated nosocomial infections in ICUs [15].

**Table 7:** Comparison of device-associated infection rates: Regional vs. National (2022) CLABSI: central line-associated bloodstream infection, CAUTI: catheter-associated urinary tract infection, VAP: ventilator-associated pneumonia, VAE: ventilator-associated event

Device-associated infection	National level (Events per 1000 device days)	Regional level (Events per 1000 device days)
CLABSI rate	2.57	4.29
CAUTI rate	1.08	0.55
VAP rate	1.53	1.17
VAE rate	4.21	3.15

## Discussion

Though surveillance has been in place for many years in the country, it is a relatively new concept in most of the hospitals in the study region. The data from the study helps in understanding the current situation and the variations that exist across the hospitals in this region. The national-level benchmarks are available, but it would be more practical to have regional-level information for more realistic and impactful surveillance and action.

The overall rate of DAI in the present study was 2.35 per 1000 patient days, which is lower than a study done by Ruo-Jie and his colleagues, which showed a rate of 5.25 per 1000 patient days [18]. Another study reported a rate of DA-HAIs of 34.1 per 1000 patient days, higher than the current research [19]. Moreover, a study done in the ICU of a university hospital in Poland reported that the crude rate of DA-HAIs was 17.13 (16.13-18.68)/1000 patient days, which was higher than our study [8].

The current study showed the majority of DAIs occurred in the bloodstream due to CLABSI, followed by CAUTI and VAP. This finding was in contrast to a study done by Ruo-Jie and colleagues, who reported that VAP was the most common DAI, followed by CAUTI and CLABSI [18]. Other studies reported the opposite result to the present study, where VAP was the most commonly diagnosed DAI, followed by CAUTI and CLABSI [8,9,20]. Similar to the result of our study, the findings of the international nosocomial infection control consortium showed CLABSI was the most common DAI [21]. The controversy over the results of the present study compared to other research could be due to the frequent use of central lines compared to other ICUs and the different timeframes of the research.

The overall CLABSI rate (4.29) in our study is higher than the Saudi national surveillance level, which was done in 2022 (2.57) [15]. This is mainly due to the high rate of 8.86 CLABSI in the neonatal ICU. As stated in another study, due to inherent reasons, the CLABSI rates are high among neonates (3.2 to 21.8 per 1000 central venous line days) [16]. Moreover, the CLABSI rate in our study is higher than the rates in NHSN (0.80), GCC (2.60), and INICC (4.93) [15]. Furthermore, even the stratified CLABSI rate in all ICUs (adult medical-surgical, neonatal ICU, and pediatric ICU) (2.71, 8.86, and 8.17, respectively) was higher than the national, GCC, and INICC [15]. In contrast to our finding, a prospective surveillance study of healthcare-associated infections reported a lower rate than ours (2.40 per 1000 patient days) [18].

As shown in Table 7, the rate of DAIs of other devices (CAUTI, VAP, and VAE) in the current study is lower or the same as compared to the national, NHSN, GCC, and INICC level rates [15]. The variation between the results of the present study and the national and international rates could be due to multiple factors such as healthcare provider's compliance with the infection control guidelines and patient risk factors. As this study was based on data from national surveillance, it was beyond the scope of this paper to address these issues.

As per our knowledge, this study is one of the first such studies with a focus on regional data. We expect this information to help the hospitals that have surveillance in place have clear roadmaps for their settings. The median numbers and the range of rates will also be helpful to hospitals, which will be starting the surveillance afresh.

Limitations

The research was done retrospectively, which led to limitations such as information bias, selection bias, and a lack of planned data. The other limitation was the short duration of the study. We plan to extend the study period for at least three to five years, conduct the research prospectively, and study all the various variables and possible risk factors for the development of DA-HAIs.

## Conclusions

Healthcare-associated infections are a common global problem, especially in the ICUs. The present study revealed that the most common DA-HAIs are CLABSI and CAUTI in the ICUs of Al-Ahsa hospitals.

The current research establishes the base for regional hospitals for DA-HAIs and national and international enrichment databases. An extensive surveillance system has to be implemented in all ICUs in all hospitals. The possible reasons for the increased rates of DAIs can be multiple, such as staff knowledge gap, shortage of supplies, staff shortages, administrative support, etc. These issues could not be explored in detail because of the in-built limitation of a data-based study. However, we believe that educational program targeting all healthcare workers regarding the prevention and control programs of device-associated healthcare infections, especially ICU staff, has to be employed in the Al-Ahsa region and all over Saudi Arabia. Further exploration to see the possible reasons has to be done and addressed. Moreover, another comprehensive research project analyzing the surveillance data should be in place, targeting all hospitals in the Al-Ahsa region and all regions and provinces of Saudi Arabia, both governmental and private (Ministry of Health and non-Ministry of Health hospitals).
